# Characterization of Social Behaviors in caspase-3 deficient mice

**DOI:** 10.1038/srep18335

**Published:** 2016-01-19

**Authors:** Shih-Ching Lo, Kimberly Scearce-Levie, Morgan Sheng

**Affiliations:** 1 Department of Neuroscience, Genentech Inc., South San Francisco, California, United States of America

## Abstract

Impaired social interaction is a defining feature of autism spectrum disorder, a neurodevelopmental disorder that shows a strong male preponderance in prevalence. Studies have identified neural circuits, neuromodulators and genetic factors involved in social behaviors, but mechanistic understanding of gender-specific social deficits is lacking. We report that deletion of the caspase-3 gene, encoding a protease with functions in apoptosis and neural plasticity, alters specific social behaviors in male mice, while leaving females unaffected. Casp3^−/−^ mice showed normal behavioral responses to olfactory cues from food, neutral chemical and biological sources. Both Casp3^−/−^ males and females displayed robust social exploration, sociability, recognition and preference for an enclosed novel mouse in the three-chamber test. However, Casp3^−/−^ males showed significantly reduced social interaction behaviors when exposed to a freely moving novel mouse, including decreased interaction time and diminished mounting. Thus caspase-3 is essential for a subset of social behaviors, but despite similar hyper-locomotion in both sexes, only male Casp3^−/−^ mice exhibited social interaction deficits, which is interesting given the male bias of autism.

Social behaviors are key to myriads of activities in social species that establish group living among males and females. In humans, social behaviors are impaired in several neuropsychiatric disorders, most notably autism spectrum disorder (ASD). A neurodevelopmental disorder with high heritability, ASD shows a strong sex bias with a 4:1 male:female ratio in prevalence^1^. Many of the identified ASD candidate genes encode for proteins that function in synapse maturation and plasticity, suggesting that maldevelopment or dysfunction of synaptic connections may contribute to social deficits^2^,^3^. Mouse genetic studies in animal models of ASD also support the importance of synaptic functions in social behaviors^4^,^5^,^6^,^7^,^8^. However, the male bias found in human ASD prevalence has not been carefully examined in these mouse models, and it is unknown how sex-specific factors impact synaptic functions and circuits underlying social behavior.

Caspase-3 is a cysteine-aspartate protease that, when activated, targets specific substrates for proteolysis. Although best known for its executioner role in apoptotic cell death, accumulating evidence points to functions for caspase-3 (and other apoptosis-related molecules) in cellular remodeling processes that are independent of apoptotic cell death, including dendritic pruning, metaplasticity, homeostatic synaptic plasticity and long-term depression (LTD)^9^,^10^,^11^,^12^,^13^,^14^. In relation to the whole organism, caspase-3 deficiency in mice (C57BL/6) affects specific aspects of cognition and behavior, particularly attention and inhibitory control, resulting in a behavioral phenotype reminiscent of attention deficit/hyperactivity disorder (ADHD)^13^.

ADHD is also more commonly diagnosed in males than females with an average ratio of 3:1. In addition, ADHD and ASD are common psychiatric comorbidities that share deficits in attention and executive control^15^. While exploring possible links between ADHD-like and autistic-like traits in genetic rodent models, we discovered that, without affecting olfaction or pheromone responses, caspase-3 deficiency impairs a subset of social behaviors in mice in a sex-dependent manner. The importance of caspase-3 in neuronal remodeling, attention control and male-specific social behavior suggests that Casp3^−/−^ mice may recapitulate some key aspects of neuropsychiatric disorders.

## Results

### Both sexes of Casp3^−/−^ mice exhibit enhanced locomotor activity

**Figure 1:**
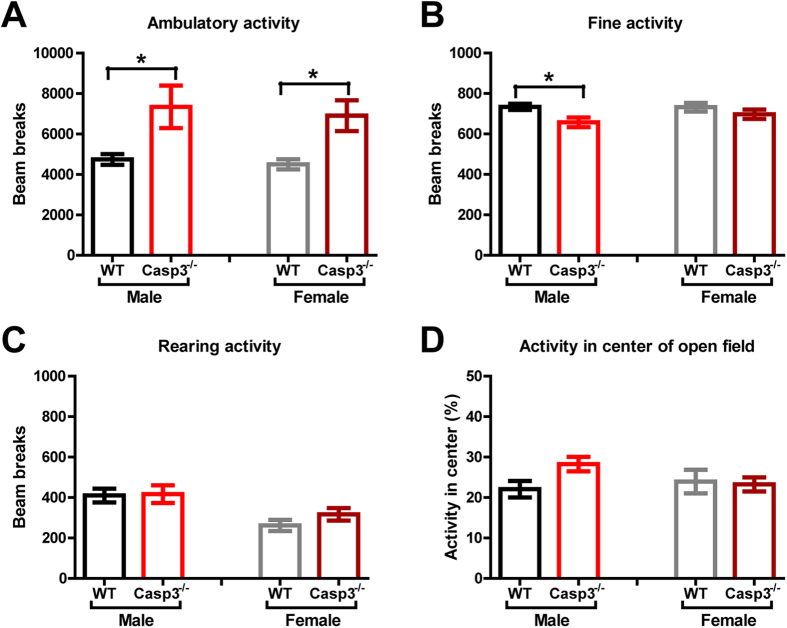
Both Casp3^−/−^ male and female mice exhibit hyperactivity in novel open field. Graphs show (**A**) total ambulatory locomotion (p = 0.004 for main genotype effect, p = 0.6220 for main sex effect, p = 0.8865 for genotype × sex, by two-way ANOVA); (**B**) total fine activity (p = 0.01 for genotype, p = 0.372 for sex, p = 0.3261 for genotype × sex, by two-way ANOVA); (**C**) total rearing (p = 0.3775 for genotype, p = 0.0006 for sex, p = 0.4874 for genotype × sex, by two-way ANOVA) in novel open-field during 1 h; (**D**) percent of locomotor activity occurring in center portion of open field during 1 h test (p = 0.2069 for genotype, p = 0.4713 for sex, p = 0.1191 for genotype × sex, by two-way ANOVA). Bar graphs show mean ± SEM for 20 WT males, 20 Casp3^−/−^ males, 20 WT females and 19 Casp3^−/−^ females. *p < 0.05 for genotype effect within each sex, by Bonferroni post-hoc tests following two-way ANOVA with the factors of genotype and sex.

A series of behavioral experiments was first conducted to evaluate potential confounding factors for sex-dependent differences in social behavior. We have previously shown that male Casp3^−/−^ mice had no deficits in neurological reflexes or motor function and coordination, but exhibited increased locomotor activity in multiple contexts^13^. We then sought to examine in both sexes of Casp3^−/−^ mice their activity levels and behavior in an open field. Both male and female Casp3^−/−^ mice displayed higher ambulatory activity than their WT littermates of the same sex (Fig. 1A). Ambulatory activity is a measure of locomotion when the animal is moving from one location to another. In contrast, fine activity, such as grooming and sniffing, remained at similar level between female mice of each genotype, and was slightly lower in male Casp3^−/−^ mice than male WT mice (Fig. 1B). In agreement with previous studies^16^, male mice overall showed higher rearing activity than female mice (Fig. 1C; p = 0.0005 by ANOVA). Nevertheless, Casp3^−/−^ and WT mice within each sex showed comparable rearing activity (Fig. 1C). Regardless of sex, there was no significant difference between genotype groups in the proportion of activities occurring in the center of the field (Fig. 1D).

### Casp3^−/−^ mice have normal behavioral responses to social and nonsocial odors

**Figure 2:**
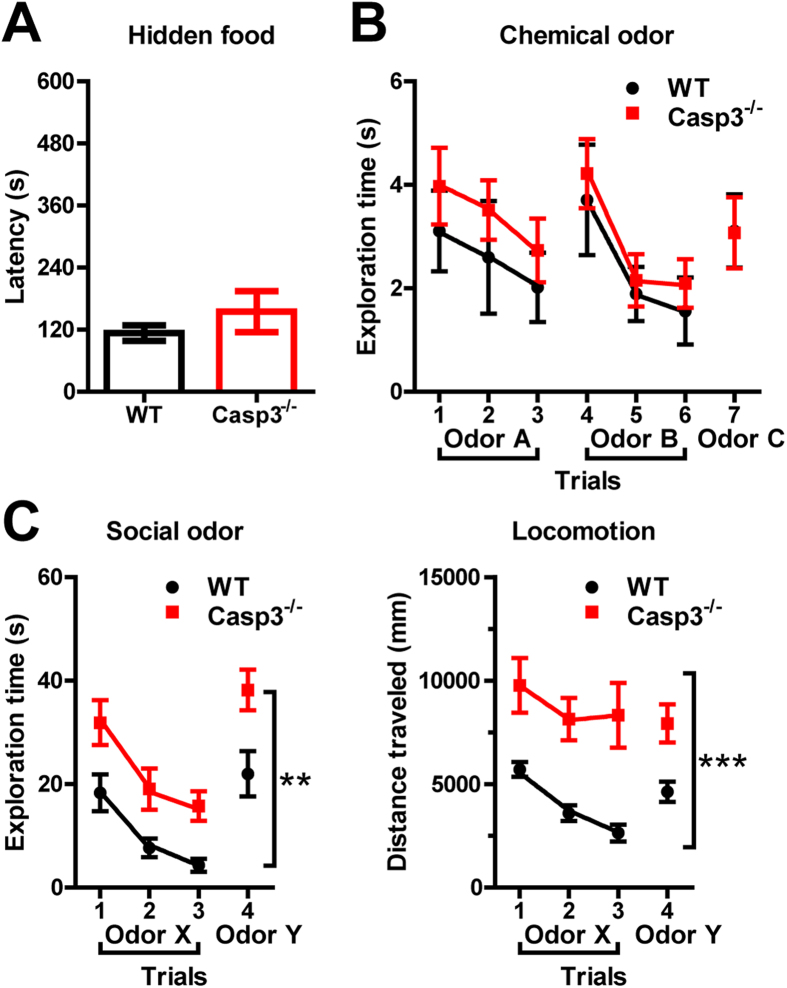
Casp3^−/−^ mice have normal behavioral responses to food, chemical and social odors. (**A**) Latency to retrieve buried food pellet in hidden food task; Bar graph shows mean ± SEM for for 21 WT males and 13 Casp3^−/−^ males; p = 0.262 for genotype effect by ANOVA. (**B**) Time spent exploring novel chemical odors over seven trials; plot shows the mean ± SEM of pooled data from 12 WT males and 6 WT females, and from 13 Casp3^−/−^ males and 6 Casp3^−/−^ females; p = 0.3202 for genotype effect by repeated measures two-way ANOVA. (**C**) Time spent exploring novel social odors (left) and distance traveled (right) over four trials; plots show the mean ± SEM for 20 WT males and 20 Casp3^−/−^ males; **p = 0.0019, ***p = 0.0005 for genotype effect by repeated measures two-way ANOVA.

Olfactory information is essential for a wide array of mouse behaviors, especially those within the social domain^17^. To confirm abilities to detect olfactory cues in Casp3^−/−^ mice, mice were first tested in the hidden food task. A piece of food pellet was buried beneath a layer of cage bedding, and animal’s ability to smell volatile odors was assessed by the time it took to successfully locate and dig out the buried food. Both Casp3^−/−^ and WT mice were equally adept at the task, with average latency of ~1–3 minutes to retrieve the hidden food (Fig. 2A).

Next, we examined the animals’ ability to distinguish different odors. A series of nonsocial odors were presented sequentially over seven consecutive trials (Fig. 2B). Both genotype groups explored a novel chemical odor similarly during trial 1, but spent less time exploring the same odor during trials 2 and 3 (habituation; Fig. 2B). When a different chemical odor was introduced during trial 4, exploration time rebounded (dishabituation) and subsequently decreased during trials 5 and 6 when the same second odor was repeated (Fig. 2B). Likewise, when a series of novel urine odors taken from same-sex mice were presented, both genotype groups showed normal habituation and dishabituation responses to these social odors (p = 0.8342 for genotype × trial interaction, by repeated measures two-way ANOVA; Fig. 2C), despite overall higher exploration of social odors in Casp3^−/−^ mice (Fig. 2C, left panel). In contrast to habituation of exploration time (Fig. 2C, left panel), Casp3^−/−^ mice had a higher level of locomotor activity that was sustained throughout all trials, while the locomotion of their WT littermates showed habituation and dishabituation that correlated with changes in their exploration time (Fig. 2C, right panel). These results suggest that olfactory recognition is not impaired in Casp3^−/−^ mice and unlikely impinged on by their hyperactivity.

### Casp3^−/−^ mice show sociability and preference for social novelty in 3-chamber assay

**Figure 3:**
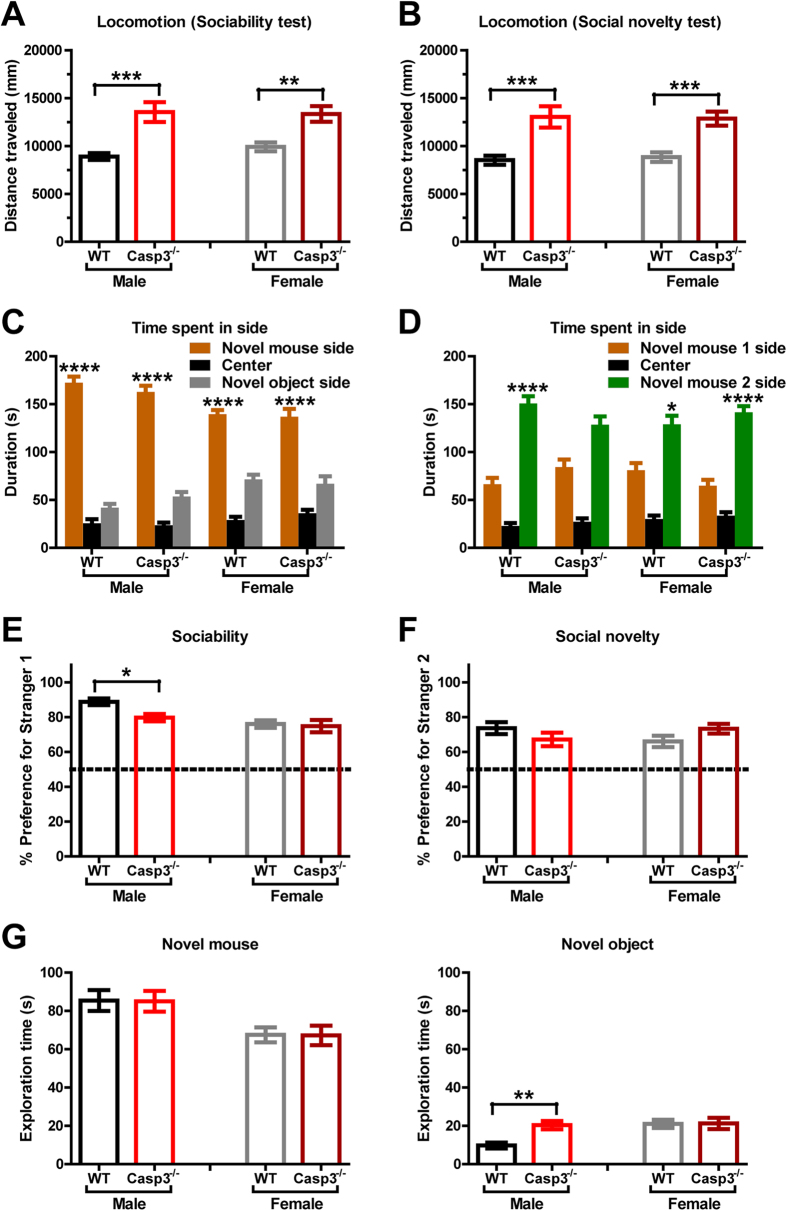
Casp3^−/−^ mice show sociability and preference for social novelty in three-chamber tests. (**A**,**C**,**E**,**G**) Sociability test. Graphs show (**A**) total distance traveled (p < 0.0001 for genotype, p = 0.5799 for sex, p = 0.4020 for genotype × sex, by two-way ANOVA); (**C**) time spent in each side chamber containing novel mouse or empty wire cup (novel object), or the center chamber (p < 0.0001 for chamber, p = 0.4877 for genotype, p = 0.0298 for sex, p = 0.3169 for chamber × genotype × sex, by three-way ANOVA); (**E**) preference for novel mouse (p = 0.0431 for genotype, p = 0.0007 for sex, p = 0.1196 for genotype × sex, by two-way ANOVA), calculated as [(time spent exploring novel mouse)/(total time spent exploring novel mouse and novel object)] × 100%; ((**G**), left) time spent exploring novel mouse (p = 0.9450 for genotype, p = 0.0006 for sex, p = 0.9992 for genotype × sex, by two-way ANOVA); ((**G**), right) time spent exploring novel object (p = 0.0190 for genotype, p = 0.0088 for sex, p = 0.0237 for genotype × sex, by two-way ANOVA). (**B**,**D**,**F**) Social novelty test. Graphs show (**B**) total distance traveled (p < 0.001 for genotype, p = 0.9251 for sex, p = 0.7376 for genotype × sex, by two-way ANOVA); (**D**) time spent in each side chamber containing novel mouse 1 (now familiar), or novel mouse 2, or the center chamber (p < 0.0001 for chamber, p = 0.2572 for genotype, p = 0.0554 for sex, p = 0.0230 for chamber × genotype × sex, by three-way ANOVA); (**F**) preference for novel mouse 2 (p = 0.9113 for genotype, p = 0.8266 for sex, p = 0.0477 for genotype × sex, by two-way ANOVA); calculated as [(time spent exploring novel mouse 2)/(total time spent exploring novel mouse 1 and novel mouse 2)] × 100%. Graphs show mean ± SEM for 20 WT males, 20 Casp3^−/−^ males, 20 WT females and 19 Casp3^−/−^ females. **p < 0.01, ***p < 0.001, ****p < 0.0001, for genotype effect within each sex for (**A**,**B**,**E**–**G**), by Bonferroni post-hoc tests following two-way ANOVA with the factors of genotype and sex.; for chamber effect between novel mouse vs. novel object in (**C**), and for novel mouse 1 vs. novel mouse 2 in (**D**), by Tukey post-hoc test following three-way ANOVA with the factors of genotype, sex and chamber side (novel mouse 1 side vs. the opposite side).

Most autistic individuals have reduced or unusual social approach^18^. We used a three-chamber apparatus to quantify sociability, social recognition and social novelty preference in Casp3^−/−^ mice^19^. In the sociability test (Fig. 3A,C,E,G), a novel same-sex gonadectomized mouse (novel mouse) was placed in one side chamber within an inverted wire cup, which prevented direct social interaction while permitting transmission of olfactory, auditory and visual stimuli. An empty wire cup (novel object) was placed in the other side chamber and served as the novel nonsocial/inanimate object. The center chamber, considered neutral, contained neither objects nor animals. Casp3^−/−^ and WT mice were allowed to freely move across all three chambers. Regardless of sex, both genotype groups spent more time in the side chamber containing the novel mouse than the other two chambers, demonstrating preference for social proximity (Fig. 3C). All groups of mice showed much greater than 50% preference for exploring the novel mouse over the empty cup (novel object), indicative of substantial degree of sociability (Fig. 3E). Female mice of either genotype had comparable sociability. Male Casp3^−/−^ mice showed 79% preference for the novel mouse, which is ~10% less than male WT mice which showed 88% preference for the novel mouse (p < 0.05 by Bonferroni post-hoc tests following two-way ANOVA with the factors of genotype and sex; Fig. 3E). Deeper analysis revealed that this difference is because WT and Casp3^−/−^ male mice explored the novel mouse to similar extents (Fig. 3G, left panel), but male Casp3^−/−^ mice showed more interest in the empty cup (novel object) and explored it for ~20 seconds on average vs ~10 s for the male WT mice (Fig. 3G, right panel). This sex-dependent difference in response to novel object is unlikely to be explained by motor or activity abnormality, since both male and female Casp3^−/−^ mice had similar and sustained hyperlocomotion throughout both three-chamber tests (Fig. 3A,B).

In the social novelty test (Fig. 3B,D,F), the first mouse (Novel mouse 1) remained in one side chamber, and was now a familiar social stimulus. A new same-sex gonadectomized mouse (Novel mouse 2) was introduced in the other side chamber. All four sex and genotype groups spent more time in the chamber containing Novel mouse 2 than the other two chambers (Fig. 3D), and showed clear preference for investigating novel mouse 2 over the now familiar novel mouse 1 (Fig. 3F). These data suggest that Casp3^−/−^ mice have normal social recognition for familiar versus novel mice, as well as a preference for newer social stimuli.

### Reduced social interaction by male Casp3^−/−^ mice in free moving social interaction assay

**Figure 4:**
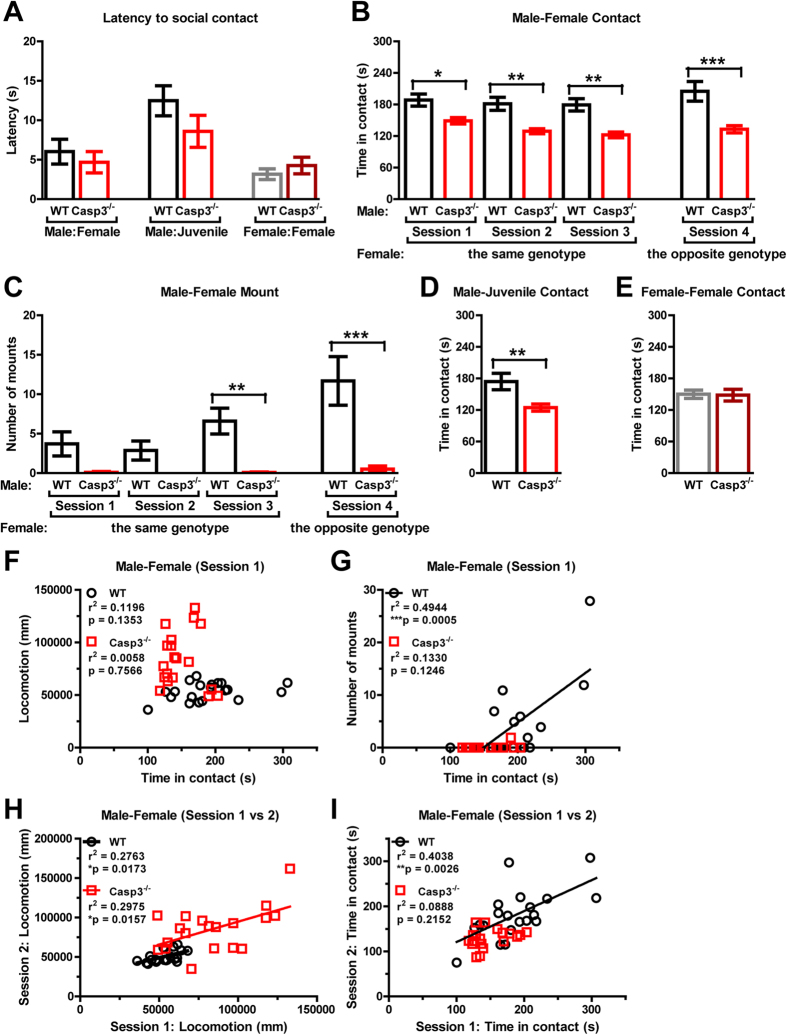
Reduced social interaction of Casp3^−/−^ male mice in freely moving reciprocal social interaction assays (**A**) Latency to make the first contact with the other mouse. (**B**) Time spent engaged in male-female social contacts during 10 min (p = 0.0001 for genotype, p = 0.0112 for session, p = 0.1111 for genotype × session, by repeated measures two-way ANOVA). (**C**) Number of male to female mountings during 10 min (p = 0.0008 for genotype, p < 0.0001 for session, p = 0.0002 for genotype × session, by repeated measures two-way ANOVA). (**D**) Time spent engaged in social contacts between a pair of adult male and juvenile C57BL/6 male mice during 10 min. (**E**) Time spent engaged in female-female social contacts during 10 min. (**F**) Relationship between time spent engaged in male-female social contacts and total distance traveled by both mice during 10 min in session 1. (**G**) Relationship between time spent engaged in male-female social contacts and number of male to female mountings during 10 min in session 1. (**H**) Correlation between total distance traveled by both mice in session 1 and session 2. (**I**) Correlation between time spent engaged in male-female social contacts in session 1 and session 2. Graphs show mean ± SEM in (**A**)-(**E**), for 20 WT males, 20 Casp3^−/−^ males, 20 WT females and 19 Casp3^−/−^ females. *p < 0.05, **p < 0.01, ***p < 0.001, for genotype effect by ANOVA in (**A**), (**D**) and (**E**), or by Bonferroni post tests following two-way ANOVA with the factors of genotype and session in (**B**) and (**C**).

Next, we examined social interaction between two freely moving mice unseparated by wire cages, a more sensitive assay for specific types of social reciprocity (Fig. 4). In session 1, we allowed same-genotype, opposite-sex pairs of adult mice to explore and interact for 10 minutes in a neutral environment to which they had been extensively habituated. Most mice exhibited immediate interest and readily approached their partners within 10 seconds and there was no difference between WT and Casp3^−/−^ mice (p = 0.5443 for genotype, by ANOVA; Fig. 4A, left). However, male-female duos of Casp3^−/−^ mice spent less time on average interacting with each other than WT male-female duos (Fig. 4B, session 1). In addition, male to female “mounting” (i.e. approaching from female’s rear and clasping female’s flanks with forepaws, usually followed by intense anogenital sniffing) was markedly reduced in Casp3^−/−^ mice (Fig. 4C, session 1). The reduced male-female contact and male-female mounting phenotypes in Casp3^−/−^ mice were confirmed in different duos of mice randomly paired up in sessions 2 and 3 (Fig. 4B,C). To determine if potential physiological differences in Casp3^−/−^ females, such as estrus cycles, might have influenced social interaction in Casp3^−/−^ mice, adult males were paired up with adult females of the opposite genotype for social interaction in session 4. Regardless of the genotype of the female partners, Casp3^−/−^ males consistently displayed less contact time than WT males, indicating that this social phenotype may be due to male factors (p = 0.0001 for main genotype effect, by repeated measures two-way ANOVA with the factors of genotype and session; Fig. 4B, sessions 1–4). Furthermore, WT males showed a progressive increase in mounting over four sessions, but Casp3^−/−^ males showed low mounting throughout all four sessions (p = 0.0008 for main genotype effect, by repeated measures two-way ANOVA; Fig. 4C). Hyperactivity is unlikely to account for these social phenotypes in Casp3^−/−^ mice, since there was minimal correlation between an animal’s locomotor activity and total social contact time (Fig. 4F). As expected, mounting in WT males positively correlated with total time they spent in contact with their partners (Fig. 4G). Interestingly, further analysis revealed a positive correlation in both genotype groups between an animal’s locomotion in two separate sessions (e.g. sessions 1 vs 2), confirming modest intra-individual variability in locomotor behavior (Fig. 4H). In comparison, a positive correlation between opposite-sex interaction time in two separate social events was only seen in WT males (Fig. 4I).

Although they took longer on average to make the first contact with juvenile males than with adult females (p = 0.0032 for latency to approach juvenile male vs. opposite sex adult, by ANOVA), Casp3^−/−^ and WT males approached juvenile males (C57BL/6) with similar latencies, usually in less than 20 seconds (p = 0.1718 by ANOVA; Fig. 4A, middle). However, adult Casp3^−/−^ males spent overall less time interacting with juvenile males (p = 0.0069 by ANOVA; Fig. 4D). In contrast, there was no genotype difference in contact time between a pair of adult females (p = 0.8982 by ANOVA; Fig. 4E), or latency to initiate interaction between female mice of the same genotype (p = 0.3864 by ANOVA; Fig. 4A, right). Together, these findings indicate that while Casp3^−/−^ females appear to be not affected, Casp3^−/−^ males have impaired social interaction with female adult mice and male juvenile mice, and show less mounting behavior towards females.

## Discussion

This study demonstrates that caspase-3 deficiency in mice results in male-specific behavioral alterations within the social domain, specifically diminished social reciprocity, as measured in a freely moving social interaction assay. The male-specific effect of caspase-3 deficiency on this social behavior is reminiscent of the strong male bias found in human ASD prevalence^1^. However, since Casp3^−/−^ males and females showed clear sociability and preference for social novelty in the three-chamber test, caspase-3 knockout mice do not recapitulate these aspects of autism. Nevertheless, this study is one of the few mouse genetic studies that carefully examined males versus females and revealed sex-dependent social deficits. A pharmacological rat model of ASD induced by prenatal exposure to anticonvulsant drug valproic acid (VPA) has been reported to show male-specific reduction in reciprocal social interaction and also in sociability in three-chamber testing^20^,^21^. Interestingly, an increase in the number of glutamatergic synapses and in postsynaptic protein expression, such as PSD-95 and CaMKII, was found in male but not female VPA treated rats^21^, raising the possibility that sex-dependent augmentation in synaptic strength may be associated with social behavior deficits in ASD. Given the key role of caspase-3 in synaptic suppression via long-term depression and homeostatic synaptic plasticity^11^,^13^, it would be interesting to investigate if there are any sex-specific effects of caspase-3 deficiency on synapses and circuit function.

Besides the male-male interaction deficit (Figs 3E and 4D), Casp3^−/−^ mice also exhibited abnormal male-female interaction, such as reduced contact time and mounting over multiple social test sessions (Fig. 4B,C). Studies have shown that pro-apoptotic proteins, including caspase-3, function in luteal regression and follicular atresia during the estrus cycle^22^,^23^. However, for Casp3^−/−^ mice, it is unlikely that female estrus abnormalities underlie poor reciprocal interaction between the opposite sexes, as evidenced by normal interaction time and vigorous mounting between Casp3^−/−^ female and WT male mice (Fig. 4B,C, session 4). Since Casp3^−/−^ mice are fertile and can produce offspring, Casp3^−/−^ males must be capable of consummatory sexual behaviors, such as mounting, intromission and ejaculation. A more detailed analysis of the appetitive phase of male sexual behaviors may further elucidate sexual arousal, motivation and goal-oriented component behaviors in Casp3^−/−^ mice. Of note, it has been reported that individuals with ASD have higher rate of asexuality, perhaps hinting at a link between reduced sexual behaviors and impaired social reciprocity in ASD^24^,^25^.

Many behavioral elements contribute to normal social behavior, including sensory perception, stress and anxiety, exploratory activity and cognition. Casp3^−/−^ males have normal vision, odor and pheromone perceptions, and learning and memory^13^ (Fig. 2). However, other factors that shape complex social behaviors may be altered in these mice. Reduction in anxiety or stress response could drive down the need for adults to engage in affiliative behaviors^26^. We did not find strong evidence for reduced anxiety-like behavior in Casp3^−/−^ males, though we did note slightly decreased fine activity (e.g. grooming) and a small but statistically insignificant increase in total activities in the center of an open field (Fig. 1B,D).

Although both Casp3^−/−^ males and females had enhanced exploratory drive in a novel place (Fig. 1A), only Casp3^−/−^ males displayed higher exploration of novel inanimate objects compared with their WT littermates of the same sex (Fig. 3G, right), perhaps analogous to the tendency of autistic individuals to engage in nonsocial activities such as playing with a favorite toy, rather than with other children in the room. Nevertheless, Casp3^−/−^ males had normal exploration of a novel mouse restricted in a wired cup (Fig. 3G), as well as preference for a relatively unfamiliar mouse in automated three-chamber testing (Fig. 3F), indicating that Casp3^−/−^ males are able to sense and retain social information and to recognize new social stimuli. This is distinct from social recognition or social memory deficits reported in mice lacking Fgf17, estrogen receptor, vasopressin receptors or oxytocin^27^,^28^,^29^,^30^,^31^. Interestingly, when exposed to a novel mouse in a freely moving environment, significant impairment was observed in Casp3^−/−^ males’ social responses. Although they showed social interest and readily explored their partners after a short latency (Fig. 4A), social contact duration and goal-directed social interactions, such as mounting, were substantially diminished in Casp3^−/−^ males (Fig. 4B–D). These findings suggest that social exploratory drive is not necessarily sufficient to achieve normal social reciprocity, which perhaps demands additional control of attention. Indeed, Casp3^−/−^ male mice have disrupted reorienting and executive processes of attention control^13^ and they model important aspects of human ADHD^13^. Further characterization of female vs. male Casp3^−/−^ mice for their attention processes in five-choice serial reaction time task may clarify the degree to which attention deficits contribute to sex-dependent impairment in social interactions.

## Methods

### Animals and general procedures

Casp3^−/−^ founder mice were purchased from the Jackson Laboratory (stock #: 006233; Yale University, laboratory of R. Flavell). Mice were backcrossed for at least 8 generations to C57BL/6. Heterozygous mice were crossed to generate Casp3^−/−^ mice and age-matched wild-type littermates. Mice were housed in a specific pathogen-free barrier facility on a 14-h/10-h light/dark cycle. Group housing was maintained throughout the study. Food and water were freely available, except where noted. Behavioral testing occurred between 08:00 and 18:00. Male and female mice were used at the same time, except where noted in Figure Legend (see Fig. 2A–C). Mice were subjected to handling habituation prior to behavioral testing. All equipment was thoroughly cleaned with 70% ethanol and water wipes between testing of individual mice to standardize odors, except where noted. Experimenters were blind to genotype. All experiments were approved by the Genentech Animal Care and Use Committee, and followed the National Institutes of Health *Guide for the Care and Use of Laboratory Animals*.

### Open Field

Mice (6 month of age) were placed in a novel open chamber (40.6 × 40.6 × 38.1 cm) made of clear plastic for 1 hour. The center region size was designated as 20 × 20 cm^2^, and the remainder of the chamber was considered periphery. Locomotor activities were monitored by a 16 × 16 photobeam system and analyzed by an automated tracking program (PAS-Open Field, San Diego Instruments).

### Hidden food test

Mice (6 month of age) were food deprived overnight prior to the test. During tests, a food pellet was buried underneath double cage bedding (bed-o’cobs) to offer a purely olfactory cue in a fresh mouse cage. Each trial started when a mouse was placed in the cage, and the time taken for the mouse to dig out the food pellet was recorded for up to a maximum of 10 minutes. Two trials were given with 4–5 hours in between, and the shorter latency between the two trials was analyzed.

### Odor Habituation and Dishabituation

Mice (8 month of age) were habituated to an empty arena (40 × 40 × 35 cm) prior to the experiment. The chemical odorants cineole (Sigma), limonene (Sigma), and peppermint (nowfoods.com) were used as novel odors in a pseudorandom order. Odors were introduced to the mice by applying 50 μl oil or extracts of each odorant onto a wood block (3.2 × 3.2 × 3.2 cm; BioServ, product # K3511) whose exact duplicates had been introduced to the mice in their home cages prior to the experiment. A single scented wood block was placed in the center of the arena, and the mice were free to explore for 3 minutes. Then, the wood block was removed, and the mouse was left in the arena for 2 minutes. An exact duplicate of the wood block applied with the same odorant was reintroduced to the arena for 3 minutes. This procedure was repeated for a total of three presentations. On the fourth trial, a different odorant was introduced, and the sequence of three presentations was repeated. The third different odorant was introduced in the final trial. Exploration of an odorant was defined as climbing on the scented block, or as when a mouse oriented toward the scented block with the distance between the nose and the block less than 1 cm. The time spent exploring an odorant was recorded from a camera mounted overhead, and analyzed by an automated tracking program (TopScan, Clever Sys Inc.), for the first 30 seconds of each trial. The same protocol was used to assess social odor (pheromone) recognition for a different cohort of mice (6 month of age). The social odor stimuli were wood blocks that had been placed and remained in the cages of a different cohort of group-housed adult male mice (C57BL/6), and soaked in urine from these mice. Odors X and Y were urine odors from different cages of mice in the same cohort (see Fig. 2C). The time spent exploring a urine soaked block was analyzed from 4 consecutive 3-minute presentations conducted as described.

### Three-chambered social approach tests

The social approach apparatus was an open-topped box made of acrylic (63 cm L × 42 cm W × 23 cm H), and divided into three chambers with two clear acrylic walls. Dividing walls had retractable doorways allowing access into each chamber. The wire cup used to contain the stranger mice was made of cylindrical chrome bars spaced 1 cm apart (10 cm H; bottom diameter: 10 cm). Test mice (9 month of age) were confined in the center chamber at the beginning of each phase. To initiate each 10-minute phase, the doorways to the side chambers were opened, and the mice were allowed to explore freely. During the habituation phase, each of the two side chambers contained an inverted empty wire cup. During the sociability phase, an unfamiliar mouse (novel mouse 1) was enclosed in one of the wire cup in a side chamber. The location of the novel mouse 1 alternated between the two side chambers across test mice. During the social novelty phase, a new unfamiliar mouse (novel mouse 2) from a different cage than novel mouse 1 was enclosed in the wire cup that had been empty during the sociability phase. Exploration of an enclosed mouse or a wire cup was defined as when a test mouse oriented toward the cup with the distance between the nose and the cup less than 1 cm, or as climbing on the cup. The time spent in each chamber and time spent exploring enclosed novel mice or empty cups (novel objects) were recorded from a camera mounted overhead, and analyzed by an automated tracking program (TopScan, Clever Sys Inc.), for the first 4 minutes of each session. Adult A/J mice (either castrated males or ovariectomized females) were purchased from Jackson Laboratory and used as stranger mice. All stranger mice were habituated to being enclosed in inverted wire cups in the three chamber apparatus for 15 minutes daily on two consecutive days prior to the experiment.

### Reciprocal social interaction test

Mice (10 month of age) were habituated to a larger cage (26 × 48.3 × 20.3 cm) for 90 minutes daily on two consecutive days prior to the experiment. During testing, two mice were placed together in a fresh cage for 10 minutes. The time spent in contact and mounting behavior were recorded from a camera mounted overhead, and analyzed by an automated tracking program (TopScan, Clever Sys Inc.). The time spent in contact was determined based on proximity of the paired mice in TopScan. Sessions 1–4 were given with 5–7 days in between. One male and one female mouse of the same genotype were randomly paired up in sessions 1–3. In session 4, one male and one female mouse of the opposite genotype were randomly paired up (see Fig. 4B,C). One adult male of either genotype and one juvenile mouse (wild-type male) were randomly paired up in male-juvenile interaction test (see Fig. 4A,D). Juvenile male C57BL/6 mice (4 weeks of age) were purchased from Charles River Laboratories. Two female mice of the same genotype were randomly paired up in female-female interaction test (see Fig. 4A,E)

## Additional Information

**How to cite this article**: Lo, S.-C. *et al*. Characterization of Social Behaviors in caspase-3 deficient mice. *Sci. Rep.* **6**, 18335; doi: 10.1038/srep18335 (2016).
